# Online Cost-Effectiveness ANalysis (OCEAN): a user-friendly interface to conduct cost-effectiveness analyses for cervical cancer

**DOI:** 10.1186/s12911-020-01232-9

**Published:** 2020-09-04

**Authors:** David Moriña, José Ignacio Martí, Pedro Puig, Mireia Diaz

**Affiliations:** 1 Barcelona Graduate School of Mathematics (BGSMath), Barcelona, Spain; 2 Departament de Matemàtiques, Universitat Autònoma de Barcelona (UAB), Cerdanyola del Vallès, 08193 Barcelona, Spain; 3 Department of Econometrics, Statistics and Applied Economics, Riskcenter-IREA, Universitat de Barcelona (UB), Barcelona, Spain; 4 Unit of Infections and Cancer - Information and Interventions (UNIC-I&I), Cancer Epidemiology Research Program (CERP), Catalan Institute of Oncology (ICO)-IDIBELL, Barcelona, Spain; 5 Centro de Investigación Biomédica en Red (CIBERONC), Barcelona, Spain

**Keywords:** Cervical cancer screening, Markov chain model, Cost-effectiveness, Online tool, cancer prevention, Decision making

## Abstract

**Background:**

Most cost-effectiveness analyses in the context of cervical cancer prevention involve the use of mathematical models to simulate HPV infection, cervical disease and prevention strategies. However, it is common for professionals who would need to perform these analyses to not be familiar with the models. This work introduces the *Online Cost-Effectiveness ANalysis* tool, featuring an easy-to-use web interface providing health professionals, researchers and decision makers involved in cervical cancer prevention programmes with a useful instrument to conduct complex cost-effectiveness analyses, which are becoming an essential tool as an approach for supporting decision-making that involves important trade-offs.

**Results:**

The users can run cost-effectiveness evaluations of cervical cancer prevention strategies without deep knowledge of the underlying mathematical model or any programming language, obtaining the most relevant costs and health outcomes in a user-friendly format. The results provided by the tool are consistent with the existing literature.

**Conclusions:**

Having such a tool will be an asset to the cervical cancer prevention community, providing researchers with an easy-to-use instrument to conduct cost-effectiveness analyses.

## Background

Health care resources are scarce and therefore, their efficient allocation is a priority for policy-makers. To guide the process of decision making in this context, cost-effectiveness analysis is an essential tool. When the available prevention strategies are multiple and potentially synergic, as occurs in the context of cervical cancer, cost-effectiveness analysis becomes critical to ensure the optimal allocation of resources [1].

Cervical cancer and other cancer and noncancer diseases are caused by or related to human papillomavirus (HPV), a common sexually transmitted infection [2]. In fact, it is estimated that more than 80% of sexually active men and women will acquire an HPV infection by age 45 years [3]. HPV infections are asymptomatic in most cases, although some can lead to the formation of cervical abnormalities called cervical intraepithelial neoplasia (CIN), which can lead to cervical cancer. This cancer is one of the most prevalent cancers among women worldwide, especially in less developed countries [4]. Cervical cancer can be prevented by means of screening to find precancerous lesions (secondary prevention) -so they can be treated- or by HPV vaccination (primary prevention) to prevent infection from some of the most frequent high-risk types. Several countries are implementing a combined prevention strategy including both vaccination and screening, which is recommended on the basis of current evidence, although details may vary. The World Health Organization (WHO) recommends that countries should implement HPV vaccination in preadolescent girls if it is affordable, cost-effective, and sustainable and that achieve the highest possible coverage [5]. However, there are still many challenges faced by cervical cancer prevention programmes [6]. Currently, most cost-effectiveness analyses focused on the evaluation of cervical cancer prevention strategies are conducted by means of mathematical models that simulate the natural history of HPV and cervical cancer. These complex models must integrate data from various sources and should be effectively calibrated to health targets. The tool presented in this work is based on a Markov model that simulates the natural history of HPV infection and subsequent cervical disease. The model computes relevant health and economic outcomes as cases averted, life expectancy (LE) from 11 years, reduction in the lifetime risk of CC, life years saved, quality-adjusted life years (QALYs), net health benefits, and lifetime costs. In this paper we present OCEAN, a new easy to use web application that allows for cost-effectiveness analyses based on a prespecified mathematical model to be run. This model is described in detail in [7]⁠. The tool and a video tutorial illustrating its usage are available at https://iconcologia.shinyapps.io/HECR-OCEAN/. Despite the tool’s ease of use and design, thought to be usable for inexperienced users, the authors are available to provide guidance in case it is needed.

## Implementation

The tool is based on a previously described Markov model [7]. The details of this model are also available as supplementary material.

### The *OCEAN* tool

The tool provides an easy-to-use web interface to conduct cost- effectiveness analyses. The sample data available from the OCEAN tool, and in particular the yearly regression and progression transition probabilities between health states were extracted from a literature review [8–12]. The web interface is based on the *shiny* package [13] for R [14]. Its main screen is divided into two panels, one focused on the calibration process and the second on the cost-effectiveness analyses.

#### Calibration process

The goal of the calibration part is to provide the user with a reliable transition probability matrix adjusted to the specific setting, to feed the Markov model that will be used in the cost-effectiveness analyses. The calibration panel requires the following inputs:

- Number of simulations: Number of simulated cohorts.
- Number of simulations to keep: Number of simulated cohorts that will be kept and used in the cost-effectiveness analyses. Among all simulated cohorts, the best ones (the matrices producing the outcomes that minimize the differences with respect to target HPV infection prevalence and CC incidence). All these simulated cohorts will be used to obtain the cost-effectiveness outputs if the *Uncertainty Level* (see the inputs for the cost-effectiveness analysis in the next subsection) is not zero, and stored each in one sheet on the same file. This allows the tool to generate several estimates for each cost-effectiveness output (one per matrix) and therefore to generate and display confidence intervals.
- Percentage of change: Maximum allowed difference between two equivalent transition probability matrices.
- Mortality: Checkbox that should be ticked if mortality data are available and it is wanted to be used in the calibration process.
- Transition probability matrix: Starting values for the transition probability matrix. A general transition probability matrix will be used if no user-specific matrix is provided.
- Incidence file: Registered cervical cancer incidence for the specific setting, without any medical intervention (rate × 100,000 women). It is uploaded in a spreadsheet file and will be used as a target in the calibration process.
- Prevalence file: Registered HPV16/18 infection prevalence for the specific setting. It is uploaded in an Excel file and will be used as a target in the calibration process.
- Mortality file: Registered mortality due to cervical cancer for the specific setting (rate × 100,000 women). It is uploaded in an Excel file and will be used as a target in the calibration process if mortality is checked.

An Excel file including all calibrated matrices can be downloaded and used for the cost-effectiveness analyses. The default transition probability matrix is calibrated to Spanish data, but the users could upload their own HPV prevalence, incidence and mortality data and calibrate the transition probabilities matrix to their own settings.

#### Cost-effectiveness analysis

The cost-effectiveness panel of the tool requires the following inputs:

- Number of different scenarios: The number of scenarios to be analyzed in the current session. Note that each scenario may include none (no intervention or natural history), one or more prevention strategies (screening alone, vaccination alone or vaccination followed by screening).
- Matrix of transition probabilities between different health states without medical intervention: Transition probability matrix obtained in the previous panel or uploaded by the user. Additionally, the included default file can be used.
- Medical costs and utility coefficients: An Excel file containing the treatment direct medical and nonmedical costs, indirect costs and utility coefficients. An example can be downloaded from the tool.
- Discount rate: Discount rate to be applied to health and costs (undiscounted results can be obtained by setting the discount rate to 0).
- Uncertainty level: It is known that the results of cost-effectiveness analyses are affected by a certain degree of uncertainty at different levels. They can be reflected in the OCEAN tool by using more than one transition probability matrix to feed the Markov model. If a file with several sheets is used and the uncertainty level is set to 0, an averaged matrix will be used, and only point estimates will be reported. If the uncertainty level is set to a value *α* between 0 and 50, all the matrices are used, and the outcomes obtained from each one are recorded. Then, the mean and percentiles $\frac{\alpha }{2}$ and $1-\frac{\alpha }{2}$ for each outcome are calculated and reported.

The considered prevention strategies that can be selected for each scenario (alone or combined) include the following:

- Natural history: No prevention strategy is considered in this scenario, which reproduces the natural history of HPV infection and cervical cancer. If this option is chosen, the other options disappear.
- Screening: Several screening scenarios can be defined, depending on test (cytology, HPV DNA test or visual inspection), frequency (every 1–10 years), targeted ages, and switch age from cytology to HPV testing. The screening coverage, positive predictive value, sensitivity and costs are read from the Excel file loaded as “Scenario-specific values”. The structure of this file can be explored by downloading the example file from the tool. Screening may be organized (all women are screened with the selected frequency) or opportunistic (the screening period is variable). To set an opportunistic screening scenario, an additional Excel file specifying the proportion of women screened each period is required.
- Vaccination: Preadolescent girls are successfully vaccinated at the age of 11 years with one, two or three doses of the vaccine against HPV types 16 and 18. Efficacy and coverage are set by the user and vaccination costs are read from the Excel file loaded as “Scenario-specific values”. The structure of this file can be explored by downloading the example file from the tool. Currently, only bivalent vaccine is considered but it is planned that quadrivalent and nonavalent vaccines will be available soon as well.

An Excel file with all generated results can be downloaded, including information about the inputs used to generate those particular results.

## Results

**Fig. 1:**
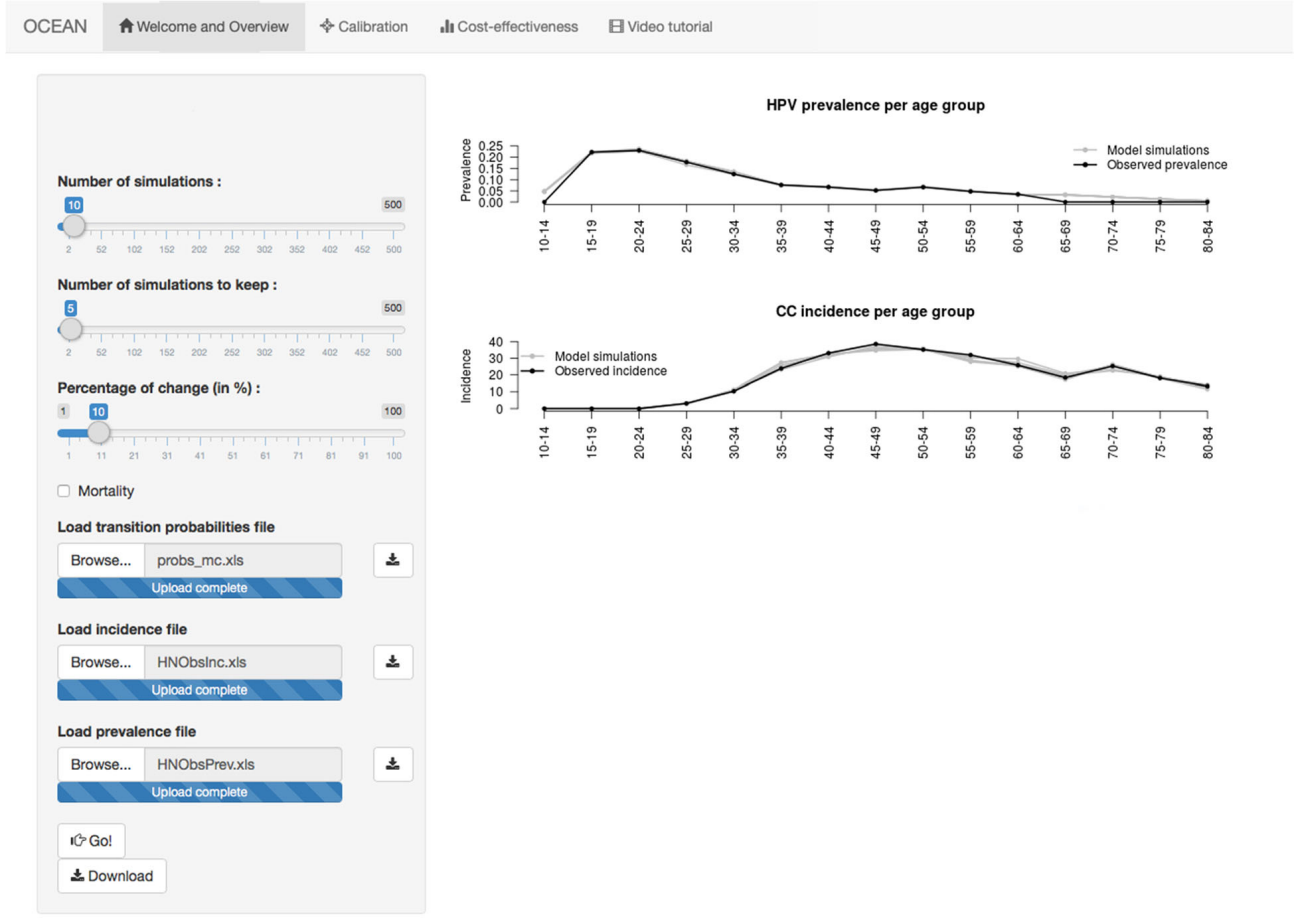
Example of the calibration panel and outputs for the age-specific cervical cancer incidence and the age-specific HPV16/18 prevalence compared to the observed data. The output is based on the 5 best-fitting (the matrices producing the outcomes that minimize the differences with respect to target HPV infection prevalence and CC incidence) simulations out of 10 demanded with a 10% change

This section reproduces the cost-effectiveness analysis reported in [7] using the OCEAN tool. The goal is to mimic some of the cervical cancer prevention strategies available in Spain, particularly comparing conventional cytology to HPV testing, with and without vaccination. All the input files used in this section are available as supplementary materials. First, we can use the calibration part of the tool to check whether the input matrix we use fits the Spanish registered data in a reasonable manner (Tables S2 and S3 show the considered HPV infection prevalence and cervical cancer incidence). Through the graphs provided by the calibration part of the *OCEAN* tool, it can be seen whether the input matrix fits the targeted values. The output calibrated matrices are also useful to introduce some random noise (determined by the percentage of change and the number of simulations to keep), allowing us to incorporate some uncertainty into the final outcomes, providing the researcher with a more realistic picture of the cost-effectiveness results. The output matrices from the calibration part can be used as input for the cost-effectiveness analysis part, where the details on the different prevention strategies considered are set. The output from the tool shows the user that the input matrix is well calibrated (Fig. 1).

Once a -or multiple- calibrated transition probability matrix that feeds the Markov model has been generated in the calibration part, it is time for the cost-effectiveness analysis. In this example, we will consider the following prevention strategies:

- Natural history: The first scenario considers no prevention strategy.
- Vaccination alone: In this scenario, preadolescent girls are successfully vaccinated at the age of 11 years with three doses of the vaccine against HPV types 16 and 18. The analysis was carried out assuming favorable vaccine with 70% efficacy and a lifelong duration of vaccine immunity to prevent cervical lesions caused by HPV 16 and 18 among uninfected women. No cross-protection against other high-risk HPV types was assumed. The uptake is set to 70%.
- Screening alone: Assuming that screening with conventional cytology starts at 25 years of age and continues until age 65, this strategy considers that primary organized HPV DNA testing is performed in women older than 30 years of age with cytology triage for positive women. Women are screened in a 5-year period. For women younger than 30 years of age, cytology is the reference test. The sensitivity and specificity of HPV DNA testing to detect CIN2+ are 90.5 and 93.0%, respectively, and 90.5 and 91.9% for cytology after a positive HPV test [15]. Screening coverage is 70%. On the basis of a study carried out in Spain, we assume that the sensitivity and specificity of cytology to detect CIN2+ are 38.2 and 97.8%, respectively [15].
- Combined vaccination and screening: In this scenario, we implement vaccination in girls aged 11 years, followed by screening according to the parameters and assumptions described previously for vaccination and screening alone.

**Fig. 2:**
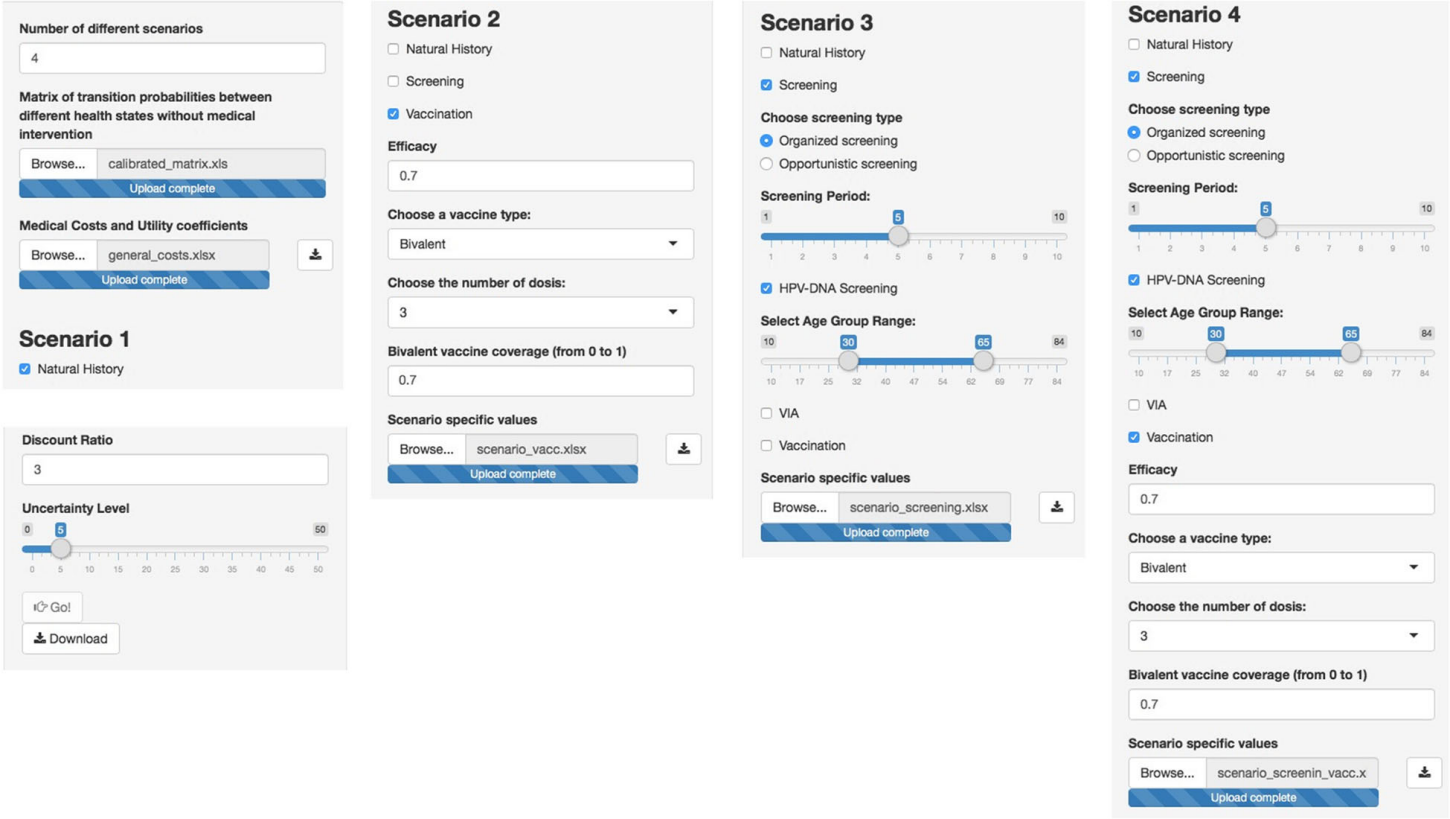
Example of inputs and parameters for four different scenarios: no intervention or natural history, vaccination alone, screening alone and combined vaccination and screening

For all the scenarios, a 3% discount rate for health outcomes and costs and a 5% uncertainty level are applied. The unitary costs used in this example are reproduced in Table S4. Figure 2 is a screenshot from the tool with the proper inputs corresponding to each described scenario.

**Fig. 3:**
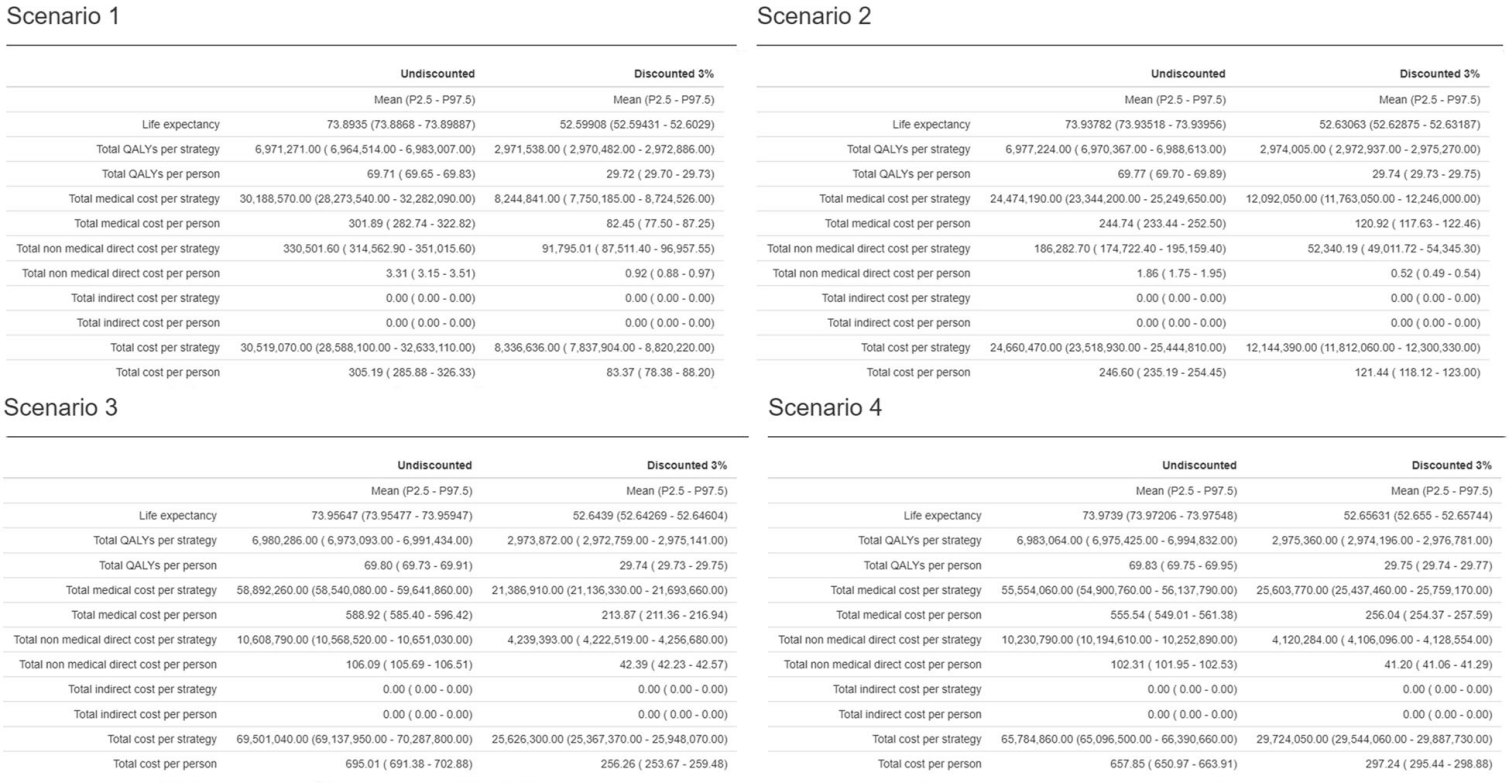
Example of output tables as reported by the tool for four different scenarios

The output tables reported by the tool can be seen in Fig. 3 and are available as supplementary material in the same format in which can be downloaded from the tool (Table S5).

The results tables from Fig. 3 show that the health and cost outcomes are very similar to those reported in [7]. A figure reproducing Fig. 3 from [7] is available as supplementary material (Fig. S2), also leading to the expected results.

## Conclusions

Carrying out complex cost-effectiveness analyses can be challenging for many researchers involved in cervical cancer prevention and for public health decision makers. The *OCEAN* tool provides an easy-to-use interface, requiring only a working internet connection and fairly available input information to allow its users to compare the most common cervical cancer prevention strategies worldwide using their specific parameters. There is a lack of robust, rigorous, accessible tools allowing users to conduct reliable cost-effectiveness analyses, as these analyses may play an important role in deciding public health policies in cervical cancer. We consider that the outputs of the *OCEAN tool* are of potential usefulness to the public health and cervical cancer prevention communities, but with some constraints.

In general, it is known that the mathematical models used in cost-effectiveness analyses are subject to uncertainty at different levels [16], and therefore, the results provided by the tool (even setting the uncertainty level over zero) must be taken with caution. In particular, the calibration process of the *OCEAN* tool does not include optimization routines, although they have been recently recommended in the literature [1] due to time limitations on the on-line interface.

Assessing the impact of uncertainty in the results, either through statistical analysis or through sensitivity analysis, is recommended by recent guidelines on health economic evaluation [17, 18]. The *OCEAN* tool facilitates the task of handling uncertainty through deterministic and probabilistic sensitivity analyses [19–21], so the implications on the cost-effectiveness analysis can be easily examined.

Several extensions of the software are currently planned. For instance, other vaccines (quadrivalent and nonavalent) will be included, and the vaccination administration period will be extended to other ages, including catch-up vaccination and male vaccination. Using a static underlying model also has some relevant limitations such as not capturing herd immunity benefits due to HPV vaccination, although this Markov model can handle complex screening strategies and it is known that static models improve the transparency and robustness of the results compared to dynamic models [22]. To overcome this issue, more sophisticated models based on microsimulation will be included in the near future.

Other HPV-related diseases (genital warts, recurrent respiratory papillomatosis and other cancer locations such as the vulva, vagina, penis, anus and oropharynx) will also be considered in upcoming versions of the tool.

### Availability and requirements

The OCEAN tool is accessible with only a working internet connection. Sample input files are available from the tool website and are ready to be used.

## Supplementary information

**Additional file 1.**

## Data Availability

The example used in this article can be reproduced by using sample input files from the tool. Additional tables and figures are available as supplementary material.
