# Supplementary material for: Online Cost-Effectiveness ANalysis (OCEAN): a user-friendly interface to conduct cost-effectiveness analyses for cervical cancer
Source: BMC Med Inform Decis Mak. 2020 Sep 4;20:211. doi: 10.1186/s12911-020-01232-9 (PMC7487926; doi:10.1186/s12911-020-01232-9)
Supplement: Supplementary file 1 — Additional file 1. [file 12911_2020_1232_MOESM1_ESM.pdf]

SUPPLEMENTARY MATERIAL

Accompanying the manuscript:

Online Cost-Effectiveness ANalysis (OCEAN): A user-friendly interface to conduct cost-effectiveness analyses

The Online Cost-Effectiveness ANalysis (OCEAN) tool is available from https://iconcologia.shinyapps.io/HECR-OCEAN. A video tutorial illustrating its usage is available from the same web address.

Underlying model

The OCEAN tool is based on a discrete-time, stochastic Markov chain model that simulates the natural history of HPV infection and cervical cancer was constructed. The basic model consists of 12 mutually exclusive and collectively exhaustive health states (a diagram is available as supplementary material, Figure S1) [healthy, HPV infection, CIN1-3 lesions, International Federation of Gynaecology and Obstetrics (FIGO) cervical cancer stages, cancer survival, cervical cancer death, and death from other causes]. Death states (both from cervical cancer and other causes) reflect country-specific female mortality stratified by age. This closed model follows a single cohort of 11-year-old girls until they reach the age of 85 years or death using equal 1-year increments, where every woman has her own probability of progressing, regressing, or remaining at the same health state. All women start model simulations as healthy and can move to the HPV-infected state by acquiring the infection with certain probability. If a woman shows clearance of the infection, she will regress to the healthy state and then, reinfection is possible. If the infection persists, the woman will move into the CIN1 state and may then progress to CIN2 and later to CIN3 and cancer or can regress and show clearance of the infection. Once in the cancer state, a woman may not regress to other health states, and instead progresses through the four stages of cancer according to the FIGO classification. A woman may die from cervical cancer if she belongs to the cancer stages or may die at any time from other non-cervical cancer cause. Nonetheless, every woman has a certain probability of developing symptoms and receiving treatment. After treatment, a woman can return to the healthy state -if she belonged to one of the CIN2-3 states- or go to the cancer survival state -if she belonged to one of the FIGO states.

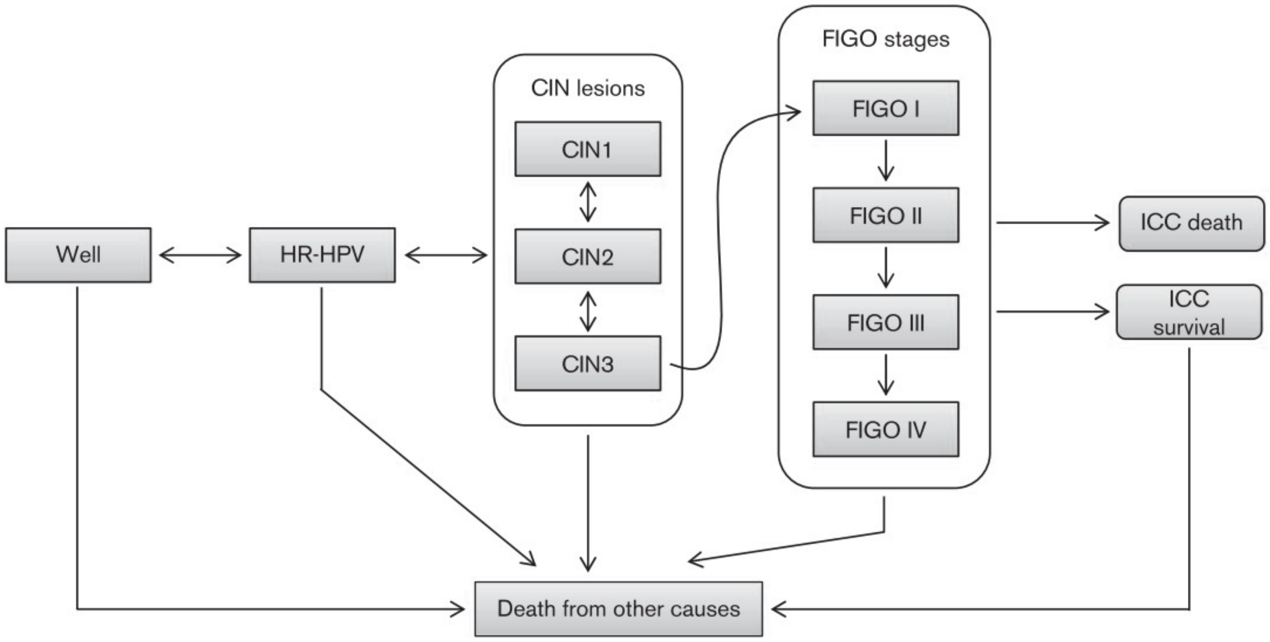

Figure S1. Diagram of the Markov model that reproduces the natural history of cervical cancer.

Age group	HPV prevalence
**10-14**	0.0000
**15-19**	0.2229
**20-24**	0.2307
**25-29**	0.1783
**30-34**	0.1250
**35-39**	0.0764
**40-44**	0.0671
**45-49**	0.0519
**50-54**	0.0663
**55-59**	0.0471
**60-64**	0.0348
**65-69**	0.0000
**70-74**	0.0000
**75-79**	0.0000
**80-84**	0.0000

Table S2. Observed age-specific high-risk HPV prevalence in Spain used in the example.

Age group	CC Incidence
**10-14**	0.000
**15-19**	0.000
**20-24**	0.000
**25-29**	3.128
**30-34**	10.400
**35-39**	23.876
**40-44**	33.084
**45-49**	38.568
**50-54**	35.160
**55-59**	31.980
**60-64**	25.824
**65-69**	18.396
**70-74**	25.304
**75-79**	18.178
**80-84**	13.344

Table S3. Observed age-specific cervical cancer incidence in Spain used in the exemple.

Strategy	Description	Direct Medical cost	Direct non-medical cost
**Vaccination**	Cost of vaccination and administration fees per dose	35.00€	0.00€
**Cytology**	Cost of cytology collection kit, staff, disposable supplies, laboratory transport, equipment, other supplies, facilities, patient transport, and cost of patient time	58.30€	17.70€
**HPV test**	Cost of HPV collection kit, staff, disposable supplies, laboratory transport, equipment, other supplies, facilities, patient transport, and cost of patient time	65.30€	17.70€
**CIN 1**	This is a weighted average of the cost of follow-up and cytology if negative result or biopsy if positive result with cytology after 6 and 12 months or follow-up with colposcopy and cytology if negative result or biopsy if positive result	256.50€	79.50€
**CIN 2-3**	Cost of treating a person who has true CIN2-3. This includes the treatment-specific staff time, supplies, equipment, hospitalization and follow-up visits and procedures as well as patient time receiving services, hospitalization and follow-up and patient transport	1,492.40€	189.60€
**FIGO I**	Cost of staging and treatment for stage 1 cancer (local cancer). This includes patient time for follow-up visits, hospitalization, patient transport, complementary procedures (pap or colposcopy)	5,521.00€	214.00€
**FIGO II**	Cost of staging and treatment for stage 2 cancer (regional cancer). This includes patient time for follow-	12,362.00€	214.00€

	up visits, hospitalization, patient transport, complementary procedures (pap or colposcopy)		
**FIGO III**	Cost of staging and treatment for stage 3 cancer (distant cancer). This includes patient time for follow-up visits, hospitalization, patient transport, complementary procedures (pap or colposcopy)	22,998.00€	214.00€
**FIGO IV**	Cost of staging and treatment for stage 4 cancer. This includes patient time for follow-up visits, hospitalization, patient transport, complementary procedures (pap or colposcopy)	33,830.00€	214.00€

Table S4. Unitary cost per woman of vaccination, screening tests, follow-up or treatment of premalignant lesions, and treatment of cervical cancer stages (costs indexed at year 2013) used in the example.

Strategy		Undiscounted.Mean	Undiscounted.P2.5	Undiscounted.P97.5	Discounted.3..Mean	Discounted.3..P2.5	Discounted.3..P97.5	V1
No intervention	Life expectancy	73.8935000	73.8868000	73.8988700	52.5990800	52.5943100	52.6029000	
	Total QALYs per strategy	6,971,271.0000000	6,964,514.0000000	6,983,007.0000000	2,971,538.0000000	2,970,482.0000000	2,972,886.0000000	
	Total QALYs per person	69.7127100	69.6451400	69.8300700	29.7153800	29.7048200	29.7288600	
	Total medical cost per strategy	30,188,570.0000000	28,273,540.0000000	32,282,090.0000000	8,244,841.0000000	7,750,185.0000000	8,724,526.0000000	
	Total medical cost per person	301.8857000	282.7354000	322.8209000	82.4484100	77.5018500	87.2452600	
	Total non medical direct cost per strategy	330,501.6000000	314,562.9000000	351,015.6000000	91,795.0100000	87,511.4000000	96,957.5500000	
	Total non medical direct cost per person	3.3050160	3.1456290	3.5101560	0.9179501	0.8751140	0.9695755	
	Total indirect cost per strategy	0.0000000	0.0000000	0.0000000	0.0000000	0.0000000	0.0000000	
	Total indirect cost per person	0.0000000	0.0000000	0.0000000	0.0000000	0.0000000	0.0000000	
	Total cost per strategy	30,519,070.0000000	28,588,100.0000000	32,633,110.0000000	8,336,636.0000000	7,837,904.0000000	8,820,220.0000000	
	Total cost per person	305.1907000	285.8810000	326.3311000	83.3663600	78.3790400	88.2022000	
	Parameter1							Discount: 3
	Parameter2							Uncertainty: 5
Vaccination	Life expectancy	73.9378200	73.9351800	73.9395600	52.6306300	52.6287500	52.6318700	
	Total QALYs per strategy	6,977,224.0000000	6,970,367.0000000	6,988,613.0000000	2,974,005.0000000	2,972,937.0000000	2,975,270.0000000	
	Total QALYs per person	69.7722400	69.7036700	69.8861300	29.7400500	29.7293700	29.7527000	
	Total medical cost per strategy	24,474,190.0000000	23,344,200.0000000	25,249,650.0000000	12,092,050.0000000	11,763,050.0000000	12,246,000.0000000	
	Total medical cost per person	244.7419000	233.4420000	252.4965000	120.9205000	117.6305000	122.4600000	
	Total non medical direct cost per strategy	186,282.7000000	174,722.4000000	195,159.4000000	52,340.1900000	49,011.7200000	54,345.3000000	
	Total non medical direct cost per person	1.8628270	1.7472240	1.9515940	0.5234019	0.4901172	0.5434530	
	Total indirect cost per strategy	0.0000000	0.0000000	0.0000000	0.0000000	0.0000000	0.0000000	
	Total indirect cost per person	0.0000000	0.0000000	0.0000000	0.0000000	0.0000000	0.0000000	
	Total cost per strategy	24,660,470.0000000	23,518,930.0000000	25,444,810.0000000	12,144,390.0000000	11,812,060.0000000	12,300,330.0000000	
	Total cost per person	246.6047000	235.1893000	254.4481000	121.4439000	118.1206000	123.0033000	
	Parameter1							Vaccination
	Parameter2							Efficacy: 0.7
	Parameter3							Vaccine Type: Bivalent
	Parameter4							N Dosis: 3
	Parameter5							Coverage: 0.7
	Parameter6							Discount: 3
	Parameter7							Uncertainty: 5
5y-HPV DNA test	Life expectancy	73.95647	73.95477	73.95947	52.64390	52.64269	52.64604	
	Total QALYs per strategy	6,980,286.00000	6,973,093.00000	6,991,434.00000	2,973,872.00000	2,972,759.00000	2,975,141.00000	
	Total QALYs per person	69.80286	69.73093	69.91434	29.73872	29.72759	29.75141	
	Total medical cost per strategy	58,892,260.00000	58,540,080.00000	59,641,860.00000	21,386,910.00000	21,136,330.00000	21,693,660.00000	
	Total medical cost per person	588.92260	585.40080	596.41860	213.86910	211.36330	216.93660	
	Total non medical direct cost per strategy	10,608,790.00000	10,568,520.00000	10,651,030.00000	4,239,393.00000	4,222,519.00000	4,256,680.00000	
	Total non medical direct cost per person	106.08790	105.68520	106.51030	42.39393	42.22519	42.56680	
	Total indirect cost per strategy	0.00000	0.00000	0.00000	0.00000	0.00000	0.00000	
	Total indirect cost per person	0.00000	0.00000	0.00000	0.00000	0.00000	0.00000	
	Total cost per strategy	69,501,040.00000	69,137,950.00000	70,287,800.00000	25,626,300.00000	25,367,370.00000	25,948,070.00000	
	Total cost per person	695.01040	691.37950	702.87800	256.26300	253.67370	259.48070	
	Parameter1							Screening
	Parameter2							Organized Screening
	Parameter3							Screening Period: 5
	Parameter4							DNA-HPV Screening
	Parameter5							DNA-HPV Screening Min Age: 30
	Parameter6							DNA-HPV Screening Max Age: 65
	Parameter7							Discount: 3
	Parameter8							Uncertainty: 5
Vaccination + 5y-HPV DNA test	Life expectancy	73.97390	73.97206	73.97548	52.65631	52.65500	52.65744	
	Total QALYs per strategy	6,983,064.00000	6,975,425.00000	6,994,832.00000	2,975,360.00000	2,974,196.00000	2,976,781.00000	
	Total QALYs per person	69.83064	69.75425	69.94832	29.75360	29.74196	29.76781	
	Total medical cost per strategy	55,554,060.00000	54,900,760.00000	56,137,790.00000	25,603,770.00000	25,437,460.00000	25,759,170.00000	
	Total medical cost per person	555.54060	549.00760	561.37790	256.03770	254.37460	257.59170	
	Total non medical direct cost per strategy	10,230,790.00000	10,194,610.00000	10,252,890.00000	4,120,284.00000	4,106,096.00000	4,128,554.00000	
	Total non medical direct cost per person	102.30790	101.94610	102.52890	41.20284	41.06096	41.28554	
	Total indirect cost per strategy	0.00000	0.00000	0.00000	0.00000	0.00000	0.00000	
	Total indirect cost per person	0.00000	0.00000	0.00000	0.00000	0.00000	0.00000	
	Total cost per strategy	65,784,860.00000	65,096,500.00000	66,390,660.00000	29,724,050.00000	29,544,060.00000	29,887,730.00000	
	Total cost per person	657.84860	650.96500	663.90660	297.24050	295.44060	298.87730	
	Parameter1							Screening
	Parameter2							Organized Screening
	Parameter3							Screening Period: 5
	Parameter4							DNA-HPV Screening
	Parameter5							DNA-HPV Screening Min Age: 30
	Parameter6							DNA-HPV Screening Max Age: 65
	Parameter7							Vaccination
	Parameter8							Efficacy: 0.7
	Parameter9							Vaccine Type: Bivalent
	Parameter10							N Dosis: 3
	Parameter11							Coverage: 0.7
	Parameter12							Discount: 3
	Parameter13							Uncertainty: 5

Table S5. Outcomes of the cost-effectiveness analyses by considered prevention strategy for the example considered.

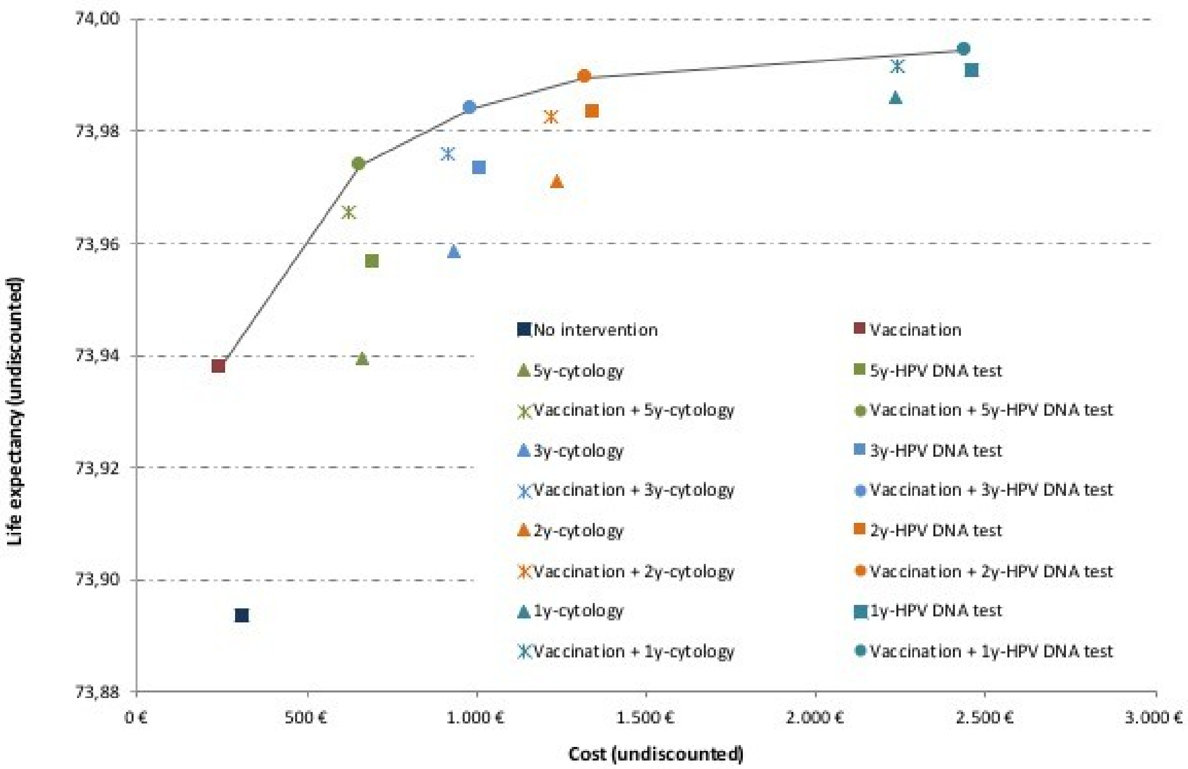

Figure S2. Cost-effectiveness frontier between undiscounted cost per person and life expectancy for the example considered. Undiscounted cost and life expectancy are drawn for all prevention strategies assessed in the described scenarios, including screening, vaccination or combined vaccination and screening.
